# Relationship between the population incidence of pertussis in children in New South Wales, Australia and emergency department visits with cough: a time series analysis

**DOI:** 10.1186/1472-6947-13-40

**Published:** 2013-03-28

**Authors:** Aaron W Cashmore, David J Muscatello, Alistair Merrifield, Paula Spokes, Kristine Macartney, Bin B Jalaludin

**Affiliations:** 1 New South Wales Public Health Officer Training Program, New South Wales Ministry of Health, North Sydney, New South Wales, Australia; 2 Population and Public Health Division, New South Wales Ministry of Health, North Sydney, New South Wales, Australia; 3 School of Public Health and Community Medicine, University of New South Wales, Kensington, New South Wales, Australia; 4 National Centre for Immunisation Research and Surveillance, Westmead, New South Wales, Australia; 5 Discipline of Paediatrics and Child Health, University of Sydney, School of Medicine, Sydney, New South Wales, Australia; 6 Centre for Research, Evidence Management and Surveillance, Sydney and South Western Sydney Local Health Districts, Liverpool, NSW, Australia

**Keywords:** Pertussis, Syndromic surveillance, Time series analysis

## Abstract

**Background:**

Little is known about the potential of syndromic surveillance to provide early warning of pertussis outbreaks. We conducted a time series analysis to assess whether an emergency department (ED) cough syndrome would respond to changes in the incidence of pertussis in children aged under 10 years in New South Wales (NSW), Australia, and to evaluate the timing of any association. A further aim was to assess the lag between the onset of pertussis symptoms and case notification in the infectious diseases surveillance system in NSW.

**Methods:**

Using routinely collected data, we prepared a daily count time series of visits to NSW EDs assigned a provisional diagnosis of cough. Separate daily series were prepared for three independent variables: notifications of cases of pertussis and influenza and ED visits with bronchiolitis (a proxy measure of respiratory syncytial virus (RSV) infection). The study period was 1/1/2007-31/12/2010. A negative binomial multivariate model was used to assess associations between the outcome and independent variables. We also evaluated the median delay in days between the estimated onset of a case of pertussis and the date the local public health authority was notified of that case.

**Results:**

When notified pertussis increased by 10 cases in one day, ED visits with cough increased by 5.2% (95% confidence interval (CI): 0.5%-10.0%) seven days later. Daily increases in the other independent variables had a smaller impact on cough visits. When notified influenza increased by 10 cases in one day, ED visits with cough increased by 0.8% (95% CI: 0%-1.7%) seven days later. When ED visits with bronchiolitis increased by 10 visits in one day, ED visits with cough increased by 4.8% (95% CI: 1.2%-8.6%) one day earlier. The median interval between estimated onset of pertussis and case notification was seven days.

**Conclusions:**

Pertussis appears to be an important driver of ED visits with cough in children aged under 10 years. However, the median delay in notification of cases of pertussis was similar to the lag in the pertussis-associated short-term increases in ED visits with cough. Elevations in RSV and influenza activity may also explain increases in the ED cough syndrome. Real time monitoring of ED visits with cough in children is therefore unlikely to consistently detect a potential outbreak of pertussis before passive surveillance.

## Background

Pertussis is an acute infection of the respiratory tract caused, in most cases, by the bacterium *Bordetella pertussis*[1,2]. Unimmunised and partially immunised infants and children are at elevated risk of contracting the infection, developing severe disease and dying of complications of pertussis [3,4]. In Australia, between 1993 and 2004, there were 17 pertussis-related deaths in infants under the age of 12 months [5].

In most developed countries, clinicians, health services managers and medical laboratory staff are required to notify public health authorities of suspected and confirmed cases of pertussis [6,7]. In Australia, pertussis is by far the most commonly notified vaccine preventable disease [5,8]. Between 2005 and 2010, the Australian age-adjusted incidence rate was 65.4 per 100,000 population [8]. Further, in the last two decades the background incidence of pertussis has steadily increased in a number of developed countries [1,9,10], including Australia [8,10]. Although a rise in testing for pertussis may have contributed to the observed increase in incidence, it is widely held that, due to factors such as variable sensitivity of available diagnostic tests during different stages of the disease, surveillance systems substantially underestimate the true population incidence of pertussis [9-11].

Reducing the spread of *B*. *pertussis* is challenging because it is a highly communicable pathogen [10] and immunity following vaccination and/or previous infection wanes after between five and 15 years [12]. Further, many infected adolescents and adults experience either mild or moderate disease. These infections often go undiagnosed and can be unwittingly transmitted to others, including susceptible children [3,13]. As a result of these and other difficulties in controlling pertussis, epidemics of the disease occur periodically, on a background of endemic circulation [5,11]. In Australia, a large outbreak started in 2008 and has continued for three years. In the Australian state of New South Wales (NSW), in the two years 2008–2009, the pertussis case notification rate was 2.7 times higher than the previous 5-year average [14].

In addition to vaccine strategies such as universal childhood immunisation [3,15], the management of cases and contacts, through activities such as case isolation and provision of antimicrobial antibiotics to cases and contacts, is a key strategy in controlling the spread of pertussis [16,17]. Such control measures are dependent on early identification and diagnosis of the infection as well as accurate and timely disease surveillance. Systems of infectious diseases surveillance based on mandatory notification of cases to public health authorities – the predominant system used in Australia, the United Kingdom, the United States and a number of other developed countries – may operate with a considerable delay [18]. Such delays have been attributed to factors such as a lag between when a case is identified by a health or laboratory professional and when that case is reported to the local public health authority [19,20]. Reporting delays can undermine the ability of public health professionals to implement control measures targeting the contacts of infectious people during the very early stages of an outbreak of a disease, including an outbreak of pertussis [19]. As a result, in recent years there has been an increase in interest in the potential of syndromic surveillance to provide early warning of outbreaks of infectious diseases [21].

Syndromic surveillance involves real time or near-real time monitoring of routinely collected health data to identify unusually high levels of a particular syndrome(s) in a population. It may provide early warning of an outbreak of disease as it allows the identification of the clinical features of infection before a diagnosis is confirmed [22,23]. Examples of sources of data that may be interrogated include: ambulance dispatches; general practitioner patient notes; and emergency department (ED) provisional diagnoses. In most systems, data are acquired electronically and statistical techniques are applied to these data to detect unusual trends in disease syndromes [22]. Compared with systems that rely on diagnostic data, syndromic surveillance can be effective in providing early warning of outbreaks of some communicable diseases [21]. To our knowledge, no studies have explored the potential of this form of surveillance to detect pertussis outbreaks.

The NSW Ministry of Health currently administers a Public Health Real-Time Emergency Department Surveillance System (PHREDSS), which enables the monitoring of visits to NSW EDs, with the aim of identifying unusual trends or patterns in visits that might signify an emerging outbreak or some other threat to public health [24]. Cough is one of 38 syndromes monitored in PHREDSS. Monitoring of this syndrome may have potential to provide early warning of outbreaks of pertussis. On the other hand, cough is a common symptom of many respiratory illnesses, including respiratory syncytial virus (RSV) and influenza. We conducted an exploratory time series analysis to assess whether the PHREDSS cough syndrome would respond to changes in the incidence of pertussis in children under the age of 10 years, and to evaluate the timing of any association. A further aim was to assess the lag between the onset of pertussis symptoms and case notification in the infectious diseases surveillance system in NSW. Time series methods have been shown to be useful in assessing whether certain syndromic data respond to changes in the incidence of related diseases in a population [18,25,26]. Establishing such correlations is needed before additional statistical techniques can be applied to syndromic data streams to identify their early warning potential.

## Methods

### Setting and data sources

Using data obtained from the NSW Emergency Department Data Collection (EDDC), we prepared a daily count time series of visits to NSW EDs assigned one of the provisional diagnoses that form the PHREDSS cough syndrome (see syndrome definitions below). The EDDC is a central repository of ED patient information. This information is collected by ED personnel in all public hospitals in NSW for the purpose of patient management. Data on visits to EDs with cough were sourced from the EDDC rather than from PHREDSS because presently more EDs contribute patient information to the EDDC. However, both systems source data from the same information systems and are therefore identical with respect to the outcome and independent variables used in our modelling.

We prepared separate daily count time series for three independent variables: notified cases of pertussis and influenza and ED visits with bronchiolitis. In NSW, notification of cases of pertussis and influenza to the state Ministry of Health is mandated under public health legislation [27]. Data on pertussis notifications (aggregated by estimated date of disease onset [28]) and influenza notifications (aggregated by specimen collection date) were obtained from the NSW Notifiable Conditions Information Management System. Because some patients wait longer than others before seeking health care, estimated date of disease onset was used in preparing the pertussis time series to better represent the population incidence of infections. Estimated date of disease onset was not used in preparing the influenza time series because, in NSW, influenza cases are exclusively notified by medical laboratory staff. Daily counts of visits to NSW EDs assigned a provisional diagnosis of bronchiolitis were used as a proxy measure of RSV incidence [25]. These data were obtained from the EDDC.

The study period was from 1 January 2007 to 31 December 2010. Only ED visits and disease notifications in children under the age of ten years were included in the various time series. This cohort was chosen as the study population because the incidence of pertussis is highest in this group [3,4]. The ED cough and bronchiolitis time series were prepared using data collected in those NSW EDs that fed patient information to the EDDC continuously during the four-year study period (N = 66). These facilities account for about 75% of all ED visits in NSW.

### Syndrome definitions

In public hospital EDs in NSW, clinicians assign each ED visit a provisional diagnosis, which is recorded in a patient management database. Not all EDs use the same database software, however. Depending on the software used, the selected provisional diagnosis is recorded as an International Classification of Diseases (ICD) code (either version 9 or 10) or a Systematized Nomenclature of Medicine – Clinical Terminology (SNOMED-CT) concept. In preparing the cough time series, we selected records of ED visits that received one of the ICD-9 or ICD-10 codes for whooping cough (ICD-9: 033. ICD-10: A37). We also selected records that received the ICD-9 or ICD-10 code for cough (786.2 and R05, respectively). Records assigned a SNOMED-CT concept that corresponds with either the ICD-9 or the ICD-10 codes for whooping cough and cough were also selected. Additional file 1 includes a complete list of the SNOMED-CT concepts that were used in selecting records.

In preparing the bronchiolitis time series, we selected records of ED visits that received one of the ICD-9 or ICD-10 codes for acute bronchiolitis (ICD-9: 466.1. ICD-10: J21). Records assigned a SNOMED-CT concept that corresponds with either the ICD-9 or the ICD-10 codes for acute bronchiolitis were also selected (Additional file 1).

### Data analysis

We used a semi-parametric generalised additive model (GAM) for count data to assess associations between ED visits with cough and three independent variables: notified cases of pertussis and influenza and ED visits with bronchiolitis. In the model we controlled for long-term trend and seasonality by fitting a non-parametric spline term for time (in days) to the outcome time series. We also controlled for the “day-of-the-week effect” and for the trend of increased ED presentations during public holidays. Such corrections allow meaningful evaluation of associations between the outcome time series and short-term changes in individual independent variables by upholding the assumption of independent observations over time. In addition, to remove remaining autocorrelation (that is, non-independence) in the model residuals, we included the previous day’s count of cough visits (a first order autoregressive term) as an additional independent variable.

For simplicity, before constructing the final model, we assessed the time lags in the relationships between the outcome and each independent variable separately. This was deemed necessary because of the potential of short-term changes in ED visits with cough to either precede or follow short-term changes in notified cases of pertussis and influenza and ED visits with bronchiolitis. The association between the outcome and each independent variable was measured separately and at different time lags (from −14 days to +14 days). Using the techniques described above (but without the first order autoregressive term), in each single variable model we controlled for medium and long-term trend as well as the “day-of-the-week effect”. The weekday was included as a categorical variable and Sunday was used as the reference category. In each independent variable, the lag that produced the strongest association with the outcome, which was taken as the lag with the relative risk furthest from unity, was identified for inclusion in the final model.

We used a GAM with the following form:

$$\begin{array}{l} \log_{e}\;\left(\mathrm{Expected}\;\left(\mathrm{daily}\;\mathrm{count}\;\mathrm{of}\;\mathrm{ED}\;\mathrm{visits}\;\mathrm{with}\;\mathrm{cough}\right)\right)\\ \;={\text{β}}_{0}+{\text{β}}_{1}\cdot \mathrm{time}+{\text{β}}_{2}\cdot \mathrm{lag}\mathrm{Pertussis}+{\text{β}}_{3}\cdot \mathrm{lagInfluenza}\\ \;+{\text{β}}_{4}\cdot \mathrm{lagBronchiolitis}+{\text{β}}_{5}\cdot \mathrm{lag}1\;\mathrm{of}\;\mathrm{ED}\mathrm{cough}\\ \;+{\text{β}}_{6}\cdot \mathrm{Monday}+{\text{β}}_{7}\cdot \mathrm{Tuesday}+{\text{β}}_{8}\cdot \mathrm{Wednesday}\\ \;+{\text{β}}_{9}\cdot \mathrm{Thursday}+{\text{β}}_{10}\cdot \mathrm{Friday}+{\text{β}}_{11}\cdot \mathrm{Saturday}\\ \;+{\text{β}}_{12}\cdot \mathrm{public}\;\mathrm{holiday}+\text{S}\left(\mathrm{time}\right) \end{array}$$

Each coefficicient, [beta], is the natural logarithm of the relative risk of the association between the respective independent variable and the outcome variable (ED visits with cough). The pertussis, influenza and bronchiolitis independent variables were scaled so that the relative risk calculated for each represented the change in the outcome associated with a 10-unit increase in the respective independent variable. Parameter estimates were converted to relative risks by raising the mathematical constant e to the power of the estimate and are expressed henceforth as percentage changes associated with a 10-unit increase. The “lag” in the names of the independent variables represents the best lag as determined by the evaluation of lagged relationships (described previously). The independent variable “lag1 of ED cough” is a first order autoregressive term of the outcome variable. It was included to control for any remaining autocorrelation in the model residuals. The day-of-week variables control for variation in use of hospital ED services by day of the week. The “public holiday” variable controls for the trend of increased use of ED services during public holidays. The “S(time)” represents a smoothing spline for time (in days), which was applied with 44 degrees of freedom to control for medium and long-term trends in the outcome variable. This amount of smoothing was found to be the minimum required to adequately limit autocorrelation in the model residuals in a comparable time series analysis [18]. The choice of 44 degrees of freedom over four years, or 11 degrees of freedom per year, effectively controls for time trend and seasonality at time scales of about one month or greater. Any remaining associations will thus be at shorter time scales, which may provide more convincing evidence of a causal relationship between time series than correlations established over longer time scales.

Because the outcome variable was daily counts of ED visits with cough, we initially used a Poisson GAM. We tested for overdispersion, or a departure from the Poisson assumption, using the sum of the squared Pearson residuals from the Poisson model and applying the Pearson chi-square goodness of fit test. This revealed significant overdispersion (Pearson χ^2^ = 1748, df = 1397, p < .001). Therefore, to account for the additional variance, we subsequently refit the GAM assuming a negative binomial distribution, which resulted in improved model fit (Pearson χ^2^ = 517, df = 1397, p = 1.000).

We assessed the quality of the final negative binomial model by examining its residuals (the standardised differences between observed and model predicted values). We also examined individual smoothing plots, residual plots (quantile-quantile plots, histograms and plots of residuals against fitted and additive predictors) and goodness of fit statistics to check GAM diagnostics. In addition, we assessed model residuals for autocorrelation (non-random behaviour suggesting poor model fit and danger of estimating biased standard errors of parameter estimates) to ensure the time trends were well controlled.

For a pertussis notification to occur, an infected person must present at a health care facility. To estimate the interval between onset of symptoms and case notification, we calculated the median delay in days between the estimated date of onset of a case of pertussis [28] and the date the local public health unit was notified of that case.

We used SAS 9.2 (SAS Institute Inc., Cary, NC, USA) for the Poisson GAM and R 2.15.1 (The R Foundation for Statistical Computing) for the negative binomial GAM. Ethical clearance to conduct this study was not required as the epidemiological data used were extracted from databases that did not contain identifying information.

## Results

Between 1 January 2007 and 31 December 2010, in NSW children under the age of ten years, there were 21,735 visits to participating EDs assigned one of the provisional diagnoses for cough. During the same period, the NSW Ministry of Health received 12,311 notifications of cases of pertussis and 4,332 notifications of cases of influenza among the study population. In addition, there were 32,120 ED visits with bronchiolitis. The highest proportion of ED visits with cough (48.6%) was in children aged between one and four years, whereas the highest proportion of notifications of cases of pertussis (54.6%) was in those aged between five and nine years (Table 1).

**Table 1:** **Emergency department (ED) visits and disease notifications in children aged under 10 years in New South Wales (NSW), Australia**^**1**^

**Age group**	**Cough ED visits**		**Pertussis notifications**		**Influenza notifications**		**Bronchiolitis ED visits**	
	**N**	**%**	**N**	**%**	**N**	**%**	**N**	**%**
**<1 year**	6,787	31.2	1,376	11.2	736	17.0	25,975	80.9
**1-4 years**	10,562	48.6	4,214	34.2	1,862	43.0	6,066	18.9
**5-9 years**	4,386	20.2	6,721	54.6	1,734	40.0	79	0.2
**Total**	21,735	100.0	12,311	100.0	4,332	100.0	32,120	100.0

1. Data sources: ED data were obtained from the NSW Emergency Department Data Collection. Disease notifications data were obtained from the NSW Notifiable Conditions Information Management System. Study period: 1 January 2007–31 December 2010.

During the study period, the median daily count of ED visits with cough among the study population was 26 (Table 2). This was a higher median daily count than those calculated for notifications of cases of pertussis (Median=5) and influenza (Median=2) and ED visits with bronchiolitis (Median=20) (Table 2).

**Table 2:** **Daily counts of emergency department (ED) visits and disease notifications: measures of central tendency and variation**^**1**^

	**Median**	**Mean**	**Standard deviation**	**Range**	**Inter-quartile range**
**Cough ED visits**	26	26.9	11.7	77.0	15.0
**Pertussis notifications**	5	8.4	10.0	54.0	11.0
**Influenza notifications**	2	12.1	40.6	416.0	6.0
**Bronchiolitis ED visits**	20	22.0	12.2	68.0	18.0

1. Data sources: ED data were obtained from the New South Wales Emergency Department Data Collection. Disease notifications data were obtained from the New South Wales Notifiable Conditions Information Management System. Study period: 1 January 2007–31 December 2010. Study population: New South Wales children aged under 10 years.

### Visual inspection of the time series

**Figure 1:**
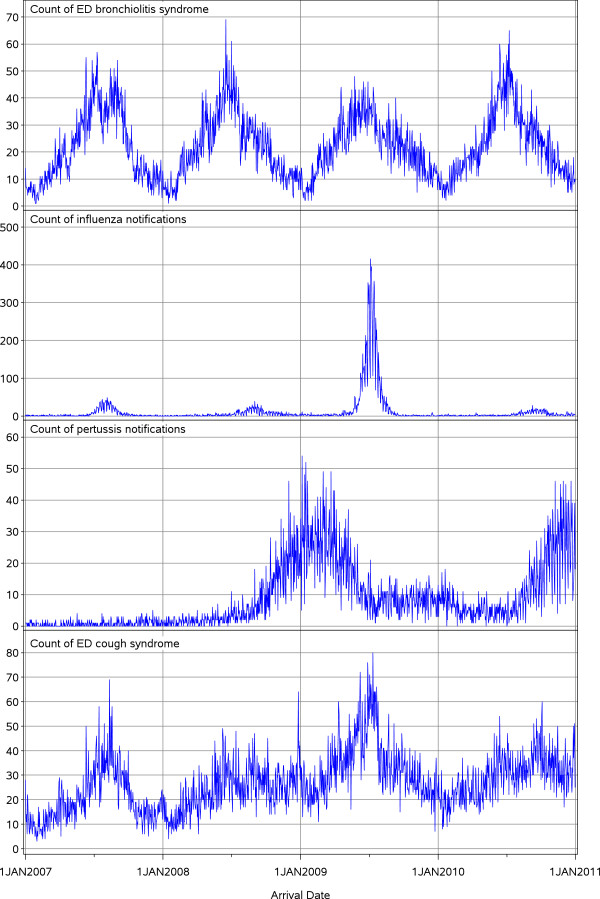
**Daily counts of emergency department (ED) visits and disease notifications in children aged under 10 years in New South Wales (NSW), Australia**^**1**^**.** 1. Data sources: ED data were obtained from the NSW Emergency Department Data Collection. Disease notifications data were obtained from the NSW Notifiable Conditions Information Management System. Study period: 1 January 2007–31 December 2010.

Visual inspection of the daily count time series revealed that ED visits with cough peaked in the Australian (southern hemisphere) winter months (June, July and August) of 2007 and 2009, and that these peaks broadly coincided with peaks in influenza and ED visits with bronchiolitis (Figure 1). In 2008 and 2010, on the other hand, ED visits with cough did not peak during winter, which appears due to substantial increases in pertussis cases in the months of September, October, November and December of those two years (Figure 1).

### Evaluation of lagged relationships

**Figure 2:**
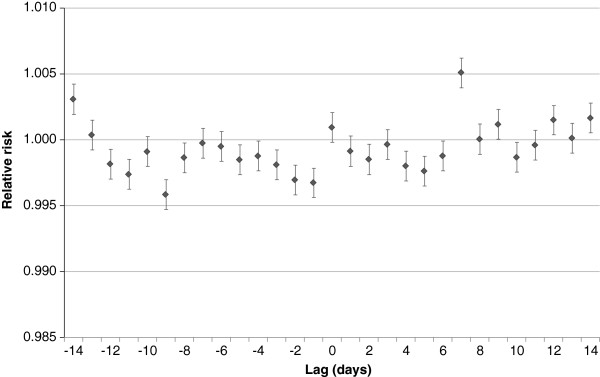
**Change in emergency department (ED) visits with cough (relative risk and 95% confidence interval) associated with a one-case increase in notifications of pertussis, lagged from −14 to +14 days: Univariate analysis**^**1**^**.** 1. A negative lag refers to notified pertussis cases (taken as date of disease onset) occurring after ED visits with cough. A positive lag refers to notified pertussis cases occurring before ED visits with cough. Relative risks refer to changes in ED visits resulting from every additional notification of a case of pertussis.

The evaluation of lagged relationships, undertaken to inform the final model, revealed that ED visits with cough were most strongly associated with notified cases of pertussis at lag +7 days. This indicates that, during the study period, short-term changes in notified cases of pertussis preceded short-term changes in ED visits with cough by one week (as an example, Figure 2 provides the results of the evaluation of the lagged relationships between the outcome and pertussis). Similarly, ED visits with cough were most strongly associated with notified cases of influenza at lag +7 days. Conversely, the outcome was most strongly associated with ED visits with bronchiolitis at lag −1 day, indicating that short-term changes in ED visits with bronchiolitis occurred 1 day after short-term changes in ED visits with cough.

### The final model – measures of association

During the study period, when notified pertussis increased by 10 cases in one day, ED visits with cough increased by 5.2% (relative risk (RR) 1.052, 95% confidence interval (CI): 1.005-1.100, p = 0.028) seven days later (Table 3). In other words, given that the median daily count of ED visits with cough during the study period was 26, a 10-case daily increase in pertussis cases was associated with a daily increase in ED visits with cough of 1.4. Daily increases in the other independent variables had a smaller impact on cough visits. When notified influenza increased by 10 cases in one day, ED visits with cough increased by 0.8% (RR 1.008, 95% CI: 1.000-1.017, p = 0.059) seven days later, and when ED visits with bronchiolitis increased by 10 visits in one day, ED visits with cough increased by 4.8% (RR 1.048, 95% CI: 1.012-1.086, p = 0.009) one day earlier (Table 3).

**Table 3:** **Final model: Change in daily emergency department (ED) visits with cough associated with 10-unit increases in independent variables**^**1,2**^

**Independent variable**	**Category**	**Parameter estimate**	**Standard error of the estimate**	**Relative risk (RR)**	**RR confidence interval (95%)**	**p-value**
**Pertussis notifications**		0.050	0.023	1.052	1.005-1.100	0.028
**Influenza notifications**		0.008	0.004	1.008	1.000-1.017	0.059
**Bronchiolitis ED visits**		0.047	0.018	1.048	1.012-1.086	0.009
**Lag 1 cough ED visits**		0.006	0.002	1.006	1.003-1.009	<.001
**Day of week**	*Sunday* (*ref.)*	0.000		1.000		
	*Monday*	0.217	0.037	1.243	1.156-1.336	
	*Tuesday*	−0.042	0.042	0.959	0.833-1.041	
	*Wednesday*	−0.097	0.038	0.908	0.842-0.979	<.001
	*Thursday*	−0.064	0.038	0.938	0.871-1.011	
	*Friday*	−0.109	0.038	0.896	0.832-0.966	
	*Saturday*	−0.084	0.038	0.919	0.853-0.991	
**Public holiday**	*Not public holiday (ref.)*	0.000		1.000		<.001
	*Public holiday*	0.287	0.061	1.333	1.182-1.502	

1. Data sources: ED data were obtained from the New South Wales Emergency Department Data Collection. Disease notifications data were obtained from the New South Wales Notifiable Conditions Information Management System. Study period: 1 January 2007–31 December 2010. Study population: New South Wales children aged under 10 years.

2. Spline term for time not included in the Table. Only the following variables were scaled by a factor of 10: Pertussis notifications; Influenza notifications; and Bronchiolitis ED visits.

**Figure 3:**
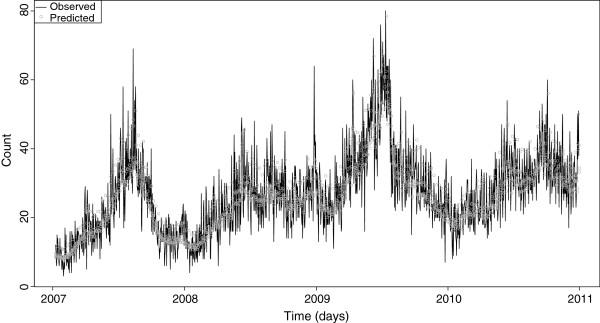
**Final model: Observed versus predicted daily counts of emergency department (ED) visits with cough in children aged under 10 years in New South Wales (NSW), Australia**^**1**^**.** 1. Data sources: ED data were obtained from the NSW Emergency Department Data Collection. Disease notifications data were obtained from the NSW Notifiable Conditions Information Management System. Study period: 1 January 2007–31 December 2010.

**Figure 4:**
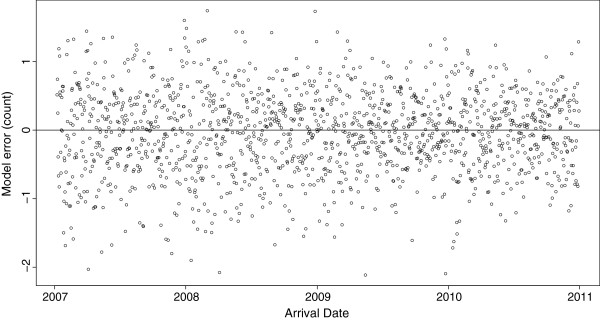
**Residual values of the final model**^**1**^**.** 1. Data sources: Emergency department data were obtained from the New South Wales Emergency Department Data Collection. Disease notifications data were obtained from the New South Wales Notifiable Conditions Information Management System. Study period: 1 January 2007–31 December 2010. Study population: New South Wales children aged under 10 years.

**Figure 5:**
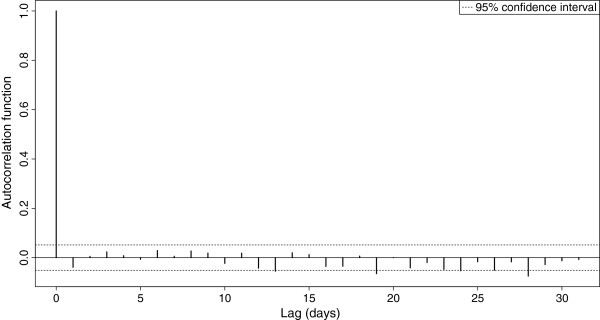
**Autocorrelation plot of the residuals of the final model**^**1**^**.** 1. Data sources: Emergency department data were obtained from the New South Wales Emergency Department Data Collection. Disease notifications data were obtained from the New South Wales Notifiable Conditions Information Management System. Study period: 1 January 2007–31 December 2010. Study population: New South Wales children aged under 10 years.

Figure 3 illustrates the observed versus fitted (predicted) daily visits to EDs with cough in NSW children under the age of ten years. Figures 4 and 5 show the final model’s residual values (errors) and the associated autocorrelation plot, respectively. The apparent randomness and lack of autocorrelation in the residuals demonstrates good model fit and control of time trends.

### Interval between disease onset and case notification

Between 1 January 2007 and 31 December 2010, the median delay between the estimated date of onset of a case of pertussis and the date the local public health unit was notified of that case was seven days.

## Discussion

Controlling for long-term trend, seasonality and the “day-of-the-week effect”, we found that short-term increases in notified cases of pertussis were independently associated with modest but statistically significant short-term increases in ED visits with cough in children under the age of 10 years. In this age group, pertussis appears to be an important, but not the sole, driver of this ED syndrome, with RSV also contributing substantially to short-term fluctuations.

Typically, in syndromic surveillance, when short-term increases in syndromes of public health significance reach a signalling threshold, epidemiologists are alerted to the increases. In NSW, if a spike in ED visits with cough among children under the age of 10 years is initially assessed by PHREDSS staff to potentially be of public health importance, state and local public health authorities are informed of the size and nature of the change. Our modelling shows that such a spike could reasonably be caused by an outbreak of pertussis. However, our findings also show that RSV activity substantially influences fluctuations in ED visits with cough among the study population and that short-term changes in notified cases of pertussis preceded short-term changes in ED visits with cough by seven days. This suggests that real time and near-real time monitoring of ED visits with cough are unlikely to consistently detect a potential outbreak of pertussis before passive surveillance, which includes mandatory reporting from numerous sources, including laboratory notification of positive tests.

There are some potential limitations of our study, which should be considered when interpreting the findings presented here. The first of these relates to the types of data used. The cough and bronchiolitis time series were prepared using ED provisional diagnoses. In NSW, in public EDs, provisional diagnoses are assigned by ED staff, most of whom are not trained medical coders. As a result, coding practices may vary among staff within EDs as well as between EDs. This introduces the potential of information bias [29] in the findings. For example, it is possible that, during the study period, some ED visits with pertussis in children under the age of 10 years were incorrectly assigned a provisional diagnosis of influenza or, in the very young, bronchiolitis. Similarly, the disease notifications data used to prepare the pertussis and influenza time series have some limitations. For example, testing for pertussis may vary among clinicians depending on their knowledge of whether *B*. *pertussis* is circulating in their community. In other words, the rate of notifications of pertussis cases is unlikely to reflect the true population incidence of the disease [9-11]. More information about the limitations of using disease notifications data in observational research can be found elsewhere [18,28].

The period of analysis in this study was four years (from 1 January 2007 to 31 December 2010). A longer time series was not possible as there was a substantial decrease in testing for and diagnosis of pertussis among NSW children in the latter months of 2006, which occurred due to problems in the performance of a commonly used serology test kit at that time [30]. A time series analysis inclusive of this period of reduced testing for and diagnosis of pertussis may have produced spurious results. On the other hand, the study period coincided with a large, on-going outbreak of pertussis in NSW, which began in 2008 [14]. This increase in disease activity may have produced a stronger relationship between ED visits with cough and notified cases of pertussis among children aged less than 10 years than would be found if a longer time series, covering periods of both high and reduced disease activity, were employed. In the future, researchers might seek to improve on this element of our study design.

Effective supplementation of passive surveillance of pertussis is needed to improve the timeliness of public health responses to a potential outbreak of the disease. A rapid response is required, not only because pertussis poses a significant threat to the health and wellbeing of communities [10], especially among vulnerable groups such as unimmunised and under-immunised children [3,4,10], but also because delays in controlling outbreaks can carry a number of additional social and economic consequences [12,31-33]. For example, a study undertaken in the 1990s in the United States found that families of children who are hospitalised with pertussis pay on average US$3,562 in direct and indirect costs [33]. Another study projected that, in the United States, between 2001 and 2010, the financial impact of adolescent pertussis alone would reach US$3.2 billion [34]. The varied costs to individuals and communities of failing to identify and respond in a timely manner to an emerging outbreak of pertussis underscore a need for further research into the utility of real time or near-real time surveillance of syndromes associated with pertussis. In the future, researchers might attempt to replicate our study among different populations. Such efforts should try to address the limitations of our design. Also, if available, additional diagnoses assigned to ED visits with pertussis or cough may be used to refine an ED cough syndrome such as the one used in PHREDSS. However, in NSW, in public EDs, nursing and medical staff are not mandated to record additional diagnoses in ED information systems. Such data are therefore frequently missing, which is why we did not include additional diagnoses in our design.

Future studies might investigate the utility of monitoring syndromic data other than ED provisional diagnoses in identifying short-term increases in pertussis cases. Liljeqvist and colleagues [35] recently developed and tested a software application capable of automated data extraction from general practitioner records. The application was found to be effective in identifying records of patients with influenza-like illness. This tool might be modified and tested for its usefulness in providing early warning of pertussis outbreaks.

## Conclusions

Pertussis appears to be an important driver of ED visits with cough in children aged under 10 years. However, the median delay in notification of cases of pertussis was similar to the lag in the pertussis-associated short-term increases in ED visits with cough. Elevations in RSV and influenza activity may also explain increases in the ED cough syndrome. Real-time monitoring of ED visits with cough in children in Australia is therefore unlikely to consistently detect a potential outbreak of pertussis before passive surveillance.

## Abbreviations

CI: Confidence interval; ED: Emergency department; EDDC: New South Wales emergency department data collection; GAM: Generalised additive model; ICD: International classification of diseases; NSW: New South Wales, Australia; PHREDSS: New South Wales public health real-time emergency department surveillance system; RSV: Respiratory syncytial virus; SNOMED-CT: Systematized nomenclature of medicine – clinical terminology

## Competing interests

We have no competing interests to declare.

## Authors’ contributions

DM and AWC conceived of the project. All authors contributed to the broader study design. DM designed and supervised the implementation of the time series analysis. AWC implemented the time series analysis and drafted the manuscript. AM assisted with data analysis. All authors critically reviewed the draft manuscript, and all authors read and approved the final version of the manuscript.

## Pre-publication history

The pre-publication history for this paper can be accessed here:

http://www.biomedcentral.com/1472-6947/13/40/prepub

## Supplementary Material

Additional file 1 **List of Systematized Nomenclature of Medicine – Clinical Terminology Concepts used in selecting records for analysis.** Full list of Systematized Nomenclature of Medicine – Clinical Terminology Concepts used in selecting records for analysis. Click here for file
