# Supplementary material for: Relationship between the population incidence of pertussis in children in New South Wales, Australia and emergency department visits with cough: a time series analysis
Source: BMC Med Inform Decis Mak. 2013 Mar 28;13:40. doi: 10.1186/1472-6947-13-40 (PMC3637193; doi:10.1186/1472-6947-13-40)
Supplement: Additional file 1 — List of Systematized Nomenclature of Medicine – Clinical Terminology Concepts used in selecting records for analysis. Full list of Systematized Nomenclature of Medicine – Clinical Terminology Concepts used in selecting records for analysis. [file 1472-6947-13-40-S1.pdf]

List of SNOMED-CT Concepts used in selecting records for analysis

Syndrome Name	SNOMED-CT Concept Identifier	SNOMED-CT Concept Name
Bronchiolitis	13089009	13089009-Adenoviral bronchiolitis (disorder)
Bronchiolitis	15199004	15199004-Acute bronchiolitis with bronchospasm (disorder)
Bronchiolitis	195734006	195734006-Acute capillary bronchiolitis (disorder)
Bronchiolitis	195737004	195737004-Acute exudative bronchiolitis (disorder)
Bronchiolitis	195738009	195738009-Obliterating fibrous bronchiolitis (disorder)
Bronchiolitis	195739001	195739001-Acute bronchiolitis due to respiratory syncytial virus (disorder)
Bronchiolitis	195740004	195740004-Acute bronchiolitis due to other specified organisms (disorder)
Bronchiolitis	195741000	195741000-Acute bronchiolitis NOS (disorder)
Bronchiolitis	196216000	196216000-[X]Acute bronchiolitis due to other specified organisms (disorder)
Bronchiolitis	233602006	233602006-Acute viral bronchiolitis (disorder)
Bronchiolitis	233603001	233603001-Acute bronchiolitis due to adenovirus (disorder)
Bronchiolitis	31886003	31886003-Bronchiolitis fibrosa obliterans (disorder)
Bronchiolitis	4120002	4120002-Bronchiolitis (disorder)
Bronchiolitis	52409006	52409006-Bronchiolitis exudativa (disorder)
Bronchiolitis	5505005	5505005-Acute bronchiolitis (disorder)
Bronchiolitis	59903001	59903001-Acute obliterating bronchiolitis (disorder)
Bronchiolitis	718004	718004-Acute bronchiolitis with obstruction (disorder)
Cough	102580004	102580004-Cough suppression (finding)
Cough	10620008	10620008-Bordetella bronchiseptica (organism)
Cough	111962006	111962006-Postural cough (finding)
Cough	11833005	11833005-Dry cough (finding)
Cough	123818007	123818007-Cough impulse impaired (finding)
Cough	135883003	135883003-Cough with fever (finding)
Cough	161422003	161422003-History of - pertussis (situation)
Cough	161927003	161927003-Night cough present (situation)
Cough	161929000	161929000-Chesty cough (finding)
Cough	161932002	161932002-Morning cough (finding)
Cough	161933007	161933007-Evening cough (finding)
Cough	161935000	161935000-Cough symptom NOS (finding)
Cough	161947006	161947006-Nocturnal cough / wheeze (finding)
Cough	162480005	162480005-Cough aggravates symptom (finding)
Cough	17986004	17986004-Barking cough (finding)
Cough	186353006	186353006-Whooping cough - other specified organism (disorder)

Syndrome Name	SNOMED-CT Concept Identifier	SNOMED-CT Concept Name
Cough	186355004	186355004-Other whooping cough NOS (disorder)
Cough	186356003	186356003-Whooping cough NOS (disorder)
Cough	187324001	187324001-[X]Whooping cough due to other Bordetella species (disorder)
Cough	187325000	187325000-[X]Whooping cough, unspecified (disorder)
Cough	19282004	19282004-Cough on exercise (finding)
Cough	20670007	20670007-Brassy cough (finding)
Cough	207066005	207066005-[D]Cough (situation)
Cough	207067001	207067001-[D]Cough syncope (situation)
Cough	225588008	225588008-Cough when swallowing
Cough	247410004	247410004-Painful cough (finding)
Cough	248592006	248592006-Character of cough (observable entity)
Cough	248593001	248593001-Cough when swallowing (finding)
Cough	248594007	248594007-Tracheal esophageal fistula cough (finding)
Cough	249596001	249596001-Cough impulse in inguinal canal (finding)
Cough	26183002	26183002-Bordetella parapertussis (organism)
Cough	263731006	263731006-Coughing (observable entity)
Cough	26484003	26484003-Bordetellosis (disorder)
Cough	271567008	271567008-Whooping cough-like syndrome (disorder)
Cough	272039006	272039006-Complaining of cough (finding)
Cough	276314008	276314008-Coughing ineffective (finding)
Cough	27836007	27836007-Pertussis (disorder)
Cough	284523002	284523002-Persistent cough (finding)
Cough	289965001	289965001-Croupy cough (finding)
Cough	300864006	300864006-Finding of cough impulse of lump (finding)
Cough	300865007	300865007-Cough impulse of mass present (finding)
Cough	300866008	300866008-Cough impulse of mass absent (finding)
Cough	300959008	300959008-Allergic cough (finding)
Cough	301235001	301235001-Finding of cough (finding)
Cough	301236000	301236000-Effective cough (finding)
Cough	301237009	301237009-Cough reflex [dup] (observable entity)
Cough	301238004	301238004-Cough reflex present (finding)
Cough	315246003	315246003-Unexplained cough (finding)
Cough	366123002	366123002-Cough reflex finding (finding)
Cough	366124008	366124008-Finding related to ability to cough (finding)
Cough	366125009	366125009-Finding related to ability to cough voluntarily (finding)

Syndrome Name	SNOMED-CT Concept Identifier	SNOMED-CT Concept Name
Cough	366126005	366126005-Finding related to ability to cough up sputum (finding)
Cough	408682005	408682005-Healthcare associated pertussis (disorder)
Cough	409596002	409596002-Non-productive cough (finding)
Cough	417850002	417850002-Respiratory tract congestion and cough (disorder)
Cough	43025008	43025008-Paroxysmal cough (finding)
Cough	46789001	46789001-Cough after eating (finding)
Cough	49727002	49727002-Cough (finding)
Cough	5247005	5247005-Bordetella pertussis (organism)
Cough	52673003	52673003-Decreased coughing (finding)
Cough	58217006	58217006-Infection due to Bordetella bronchiseptica (disorder)
Cough	59786004	59786004-Weavers' cough (disorder)
Cough	59994004	59994004-Hacking cough (finding)
Cough	62427007	62427007-Bovine cough (finding)
Cough	62548007	62548007-Nocturnal cough (finding)
Cough	62618004	62618004-Early morning cough (finding)
Cough	62731002	62731002-Increasing frequency of cough (finding)
Cough	63000007	63000007-Spasmodic cough (finding)
Cough	68154008	68154008-Chronic cough (finding)
Cough	7142008	7142008-Cough at rest (finding)
Cough	77116006	77116006-Infection due to Bordetella parapertussis (disorder)
Cough	82670009	82670009-Whooping cough due to organism other than Bordetella pertussis (disorder)
